# Acute Care Patient Portal Intervention: Portal Use and Patient Activation

**DOI:** 10.2196/13336

**Published:** 2019-07-18

**Authors:** Kumiko O Schnock, Julia E Snyder, Theresa E Fuller, Megan Duckworth, Maxwell Grant, Catherine Yoon, Stuart Lipsitz, Anuj K Dalal, David W Bates, Patricia C Dykes

**Affiliations:** 1 Division of General Internal Medicine and Primary Care Brigham and Women's Hospital Boston, MA United States; 2 Harvard Medical School Boston, MA United States

**Keywords:** patient portals, patient participation, patient activation, patient-centered care, inpatients

## Abstract

**Background:**

Patient-facing health information technology (HIT) tools, such as patient portals, are recognized as a potential mechanism to facilitate patient engagement and patient-centered care, yet the use of these tools remains limited in the hospital setting. Although research in this area is growing, it is unclear how the use of acute care patient portals might affect outcomes, such as patient activation.

**Objective:**

The aim of this study was to describe the use of an acute care patient portal and investigate its association with patient and care partner activation in the hospital setting.

**Methods:**

We implemented an acute care patient portal on 6 acute care units over an 18-month period. We investigated the characteristics of the users (patients and their care partners) of the patient portal, as well as their use of the portal. This included the number of visits to each page, the number of days used, the length of the user’s access period, and the average percent of days used during the access period. Patient and care partner activation was assessed using the short form of the patient activation measure (PAM-13) and the caregiver patient activation measure (CG-PAM). Comparisons of the activation scores were performed using propensity weighting and robust weighted linear regression.

**Results:**

Of the 2974 randomly sampled patients, 59.01% (1755/2974) agreed to use the acute care patient portal. Acute care patient portal enrollees were younger, less sick, less likely to have Medicare as their insurer, and more likely to use the Partners Healthcare enterprise ambulatory patient portal (Patient Gateway). The most used features of the acute care patient portal were the laboratory test results, care team information, and medication list. Most users accessed the portal between 1 to 4 days during their hospitalization, and the average number of days used (logged in at least once per day) was 1.8 days. On average, users accessed the portal 42.69% of the hospital days during which it was available. There was significant association with patient activation on the neurology service (*P*<.001) and medicine service (*P*=.01), after the introduction of HIT tools and the acute care patient portal, but not on the oncology service.

**Conclusions:**

Portal users most often accessed the portal to view their clinical information, though portal usage was limited to only the first few days of enrollment. We found an association between the use of the portal and HIT tools with improved levels of patient activation. These tools may help facilitate patient engagement and improve outcomes when fully utilized by patients and care partners. Future study should leverage usage metrics to describe portal use and assess the impact of HIT tools on specific outcome measures in the hospital setting.

## Introduction

### Background

The acute care setting presents challenges for patients and their care partners who often feel disengaged and disempowered [1]. The experience can be isolating and uncertain, and patients are often left out of the decision-making process [2]. Engaging patients and encouraging active participation in their care may help address these issues and has the potential to improve health outcomes as well as the quality and safety of care [3]. Health information technology (HIT) has been shown to promote patient engagement and patient-centered care [4]. Previous research by our group found that engaging patients and health care providers in the intensive care unit using patient-centered HIT tools was associated with a reduction in adverse events and improved patient satisfaction [5].

Providing patients access to their personal health records and health care information through patient portals may improve patient satisfaction, outcomes, and safety [6-9]. Given the incentives associated with providing patients access to health information through the Meaningful Use program, outpatient portals are becoming increasingly common [10]; however, the use of patient portals during acute care hospitalizations remains limited [11]. Although research on acute care patient portals is expanding [7,9,12,13], few large-scale clinical trials have been conducted, and evidence supporting their impact on improved health outcomes is currently insuf­ficient. [4,11-15].

Patient activation represents an important outcome measure. It refers to a patient’s knowledge, skills, and confidence in managing their health condition [15]. Patient activation can be an indicator of patient engagement [16]. High levels of patient activation have been associated with lower costs and better outcomes [17-19]. Patient portals may represent a mechanism to improve patient activation; however, there is limited research assessing their association with patient activation in the acute care setting. For example, a recent randomized controlled trial conducted by Masterson-Creber et al found that access to an acute care patient portal did not significantly improve patient activation [20]. Similarly, O’Leary found that the use of an acute care patient portal had no significant effect on patient activation scores [21]. Another study found that patient activation scores increased over time with the use of an acute care patient portal designed for patients undergoing hematopoietic cell transplantation, but not linearly, suggesting that a *sweet spot* of utilization may exist [22]. However, these previous studies had small sample sizes and the study results may be insufficient to characterize the association of the use of an acute care patient portal with patient activation.

### Purpose of the Study

In this study, we conducted a large-scale intervention, implementing a patient portal, along with a suite of patient and provider-facing tools, to promote patient-centered care in the acute care setting. We assessed portal usage and analyzed the association between the acute care portal and patient activation. We hypothesized that successful implementation and use of the patient portal by inpatients would result in greater knowledge of their care and increased patient activation.

## Methods

### Setting and Participants

**Figure 1:**
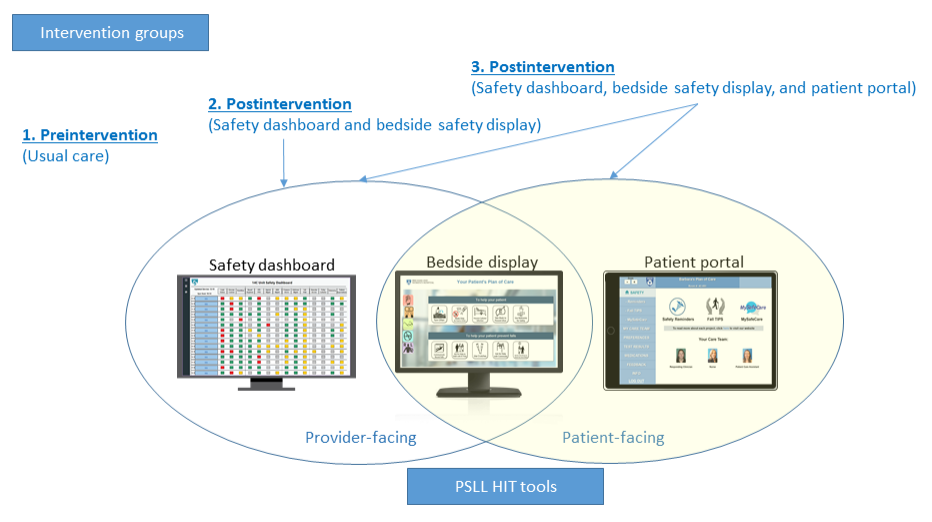
Patient safety learning laboratory and health information technology tools and intervention groups. PSLL: Patient Safety Learning Laboratory; HIT: health information technology.

The patient portal was developed and implemented as part of an Agency for Healthcare Research and Quality (AHRQ)–funded Patient Safety Learning Laboratory (PSLL) project at Brigham and Women’s Hospital (BWH), a large tertiary care center in Boston, Massachusetts. The PSLL project aimed to develop and implement a suite of HIT tools to engage patients and providers in improving quality and safety in the acute care setting. The HIT tools included a provider-facing safety dashboard [23], bedside safety display [24], and a patient portal (Figure 1). The provider-facing safety dashboard was used as a care team rounding tool and was only accessed by the health care providers [23]. The bedside safety display was both provider and patient facing. All patients were continuously exposed to the personalized bedside display monitor, whereas providers were intermittently exposed when in the patient room [24]. Randomly selected patients also received the patient portal that was a patient-facing tool accessed via a tablet computer or mobile device. These tools were implemented for an 18-month period from December 2016 to May 2018.

The PSLL tools were implemented using a randomized stepped-wedge design. Implementation of the intervention and recruitment for the patient portal began on a new inpatient unit every 1 to 2 months. In total, 6 units participated, including 3 general medicine, 1 neurology, and 2 oncology units. Patient activation was measured at 2 time points with 3 distinct groups of patients: (1) preintervention (usual care), (2) postintervention (patients exposed to the safety dashboard and bedside display), and (3) postintervention (patients exposed to the safety dashboard, bedside display, and patient portal).

### The Patient Portal

The patient portal was a Web-based application specifically designed for the acute care setting. It leveraged vendor-based (Epic Systems Inc) electronic health record (EHR) data to provide patients and families access to the real-time information and educational content needed to proactively engage in their care during hospitalization. Features of the portal included the following: personalized safety reminders and a fall prevention plan (Fall TIPS) [25]; names and photos of care team members, medication lists, laboratory test results; a method to report safety concerns (MySafeCare) [26]; and general hospital information (Multimedia Appendix 1). In December 2017, a discharge preparedness checklist was added to the portal, and in March 2018, a safety issues dashboard was added to enhance the portal content and promote patient engagement. A mobile app was also developed with the same functions and features. The user interface and content of the patient portal were developed in collaboration with patients and their care partners through participatory iterative design [5,24].

### Recruitment and Enrollment

Research staff approached randomly selected patients or their care partners (health care proxies) on study units each weekday to offer the use of the patient portal. Patients who did not speak English, were not alert and oriented, had impairments that prohibited the use of the portal, or did not have a health care proxy were excluded. All other patients on the study units and on a medicine, neurology, or oncology service were eligible to participate. Patients were offered use of the portal on a tablet computer (iPad; Apple, Inc) provided by the study or their own device for the duration of their hospital stay. The mobile version was offered beginning in December 2017, available to download as a mobile app on Apple devices. An email address (or username) was required to set up a secure account and the research staff gave a brief orientation to the portal. More than one user could be created with the patient’s permission (eg, patient and family member). The study staff provided their contact information, including an email address and phone number, for additional support. All study activities were approved by the Partners Healthcare Institutional Review Board.

### Measures and Data Analysis

We measured patient and care partner use of the portal by recording user actions in our database and leveraged previously reported measures of portal usage for comparison [7,11,20,22,27-29]. Measures included the number of visits to each page, the number of days used, length of users’ access period, and average percent of days used during the access period. Demographic characteristics of patients who enrolled and patients who declined to participate were obtained from our EHR, and differences in portal users and nonusers were compared using a Fischer exact test and robust chi-square tests [30]. Owing to a technical issue in our database, portal activity for 136 users (136/1755, 7.75%) was not recorded. We compared the patient characteristics of this group with the other enrollees’ usage data in Table 1. There were no significant differences between the 2 groups; therefore, we conducted the analysis without the missing data.

**Table 1:** Patient characteristics.

Variable		Patients that enrolled to portal (n=1755)			Patients that declined portal (n=1219)	*P* value
		Enrolled with usage data	Enrolled without usage data	*P* value		
Number of unique patients, n (%)		1619 (92.25)	136 (7.74)	—^c^	1219 (100.00)	—
Age (years), mean (SD)		56.84 (17.29)	54.96 (19.00)	.35	61.21 (16.69)	<.001
Female, n (%)		883 (54.54)	81 (59.56)	.26	646 (52.99)	.30
**Race, n (%)**						
	White	1256 (77.58)	103 (75.74)	.58	948 (77.77)	.52
	Black or African American	202 (12.48)	16 (11.76)	—	162 (13.29)	—
	Asian	28 (1.73)	4 (2.94)	—	23 (1.89)	—
	Other^a^	101 (6.24)	8 (5.88)	—	68 (5.58)	—
	Unavailable	21 (1.30)	2 (1.47)	—	10 (0.82)	—
	Declined	11 (0.68)	3 (2.21)	—	8 (0.66)	—
**Ethnicity, n (%)**						
	Hispanic or Latino	111 (6.86)	8 (5.88)	.91	67 (5.50)	.34
	Non-Hispanic	1461 (90.24)	124 (91.18)	—	1119 (91.80)	—
	Unavailable	47 (2.90)	4 (2.94)	—	32 (2.71)	—
**Primary language, n (%)**						
	English	1525 (94.19)	128 (94.12)	.95	1158 (95.00)	.16
	Spanish	26 (1.61)	2 (1.47)	—	10 (0.82)	—
	Other	22 (1.36)	2 (1.47)	—	11 (0.90)	—
	Unavailable	46 (2.84)	4 (2.94)	—	40 (3.28)	—
Charlson score, mean (SD)		2.39 (2.80)	2.26 (2.66)	.76	3.23 (3.02)	<.001
**Insurance, n (%)**						
	Private	777 (47.99)	73 (53.68)	.63	498 (40.85)	<.001
	Medicaid	161 (9.94)	9 (6.62)	—	106 (8.70)	—
	Medicare	626 (38.67)	49 (36.03)	—	572 (46.92)	—
	Self-pay	36 (2.22)	3 (2.21)	—	27 (2.21)	—
	Other	19 (1.17)	2 (1.47)	—	16 (1.31)	—
Median income by zip code, mean (SD)		71,165.68 (26,541.36)	72,658.02 (28,874.67)	.56	72,054.16 (27,478.91)	.44
Patient Gateway users, n (%)		286 (17.67)	32 (23.53)	.09	168 (13.78)	.002
Length of stay^b^, mean (SD)		8.85 (8.99)	5.83 (5.83)	<.001	8.61 (8.44)	.63

^a^Includes *Hispanic or Latino* as a race choice.

^b^First admission to time of first enrollment or decline.

^c^No data.

We used the short form of the patient activation measure (PAM-13) [31] to assess patient activation. For patients who could not participate in the PAM-13 survey, we surveyed their caregivers using the caregiver version of PAM-13 (caregiver patient activation measure [CG-PAM]) [32] to assess their activation. The PAM-13 and CG-PAM are validated 13-item instruments, with scores ranging from 0 to 100, measuring patient skill, knowledge, and confidence for self-management of health conditions [31,32]. The PAM-13 has been shown to be reliable in both outpatient and inpatient settings [33]. The PAM-13 (or CG-PAM) was administered to a random sample of patients—including both patient portal users and nonportal users—across all study units before and after the implementation of the PSLL intervention. Research staff approached patients at multiple times throughout the day using randomized lists. In all cases, patients were approached approximately one to two days before their discharge. PAM surveys were anonymous and only identified on the unit level.

The preintervention period began 3 months before the implementation of the intervention (September-November 2016), and the postintervention period occurred for 18 months after the first implementation (December 2016-May 2018). Within each of the 3 services (medicine, oncology, and neurology), we compared PAM scores across the 3 groups (see Figure 1): (1) preintervention (usual care), (2) postintervention (safety dashboard and bedside display), and (3) postintervention (safety dashboard, bedside display, and patient portal). We noted that the goal was not to compare PAM scores across services but to compare the scores before and after the intervention on the service level. To assess whether patient-reported characteristics could confound PAM score differences within each of the 3 services, we used a Fisher exact test to compare categorical variables across the 3 intervention groups and we used robust chi-square tests [30] (which do not assume normality) to compare continuous variables across the 3 groups (see Multimedia Appendix 2). Differences in patient characteristics among the 3 groups were controlled for using a weighted propensity score analysis [34]. Within each of the 3 services, the propensities of patients being in the 3 groups were estimated using a multinomial logistic regression model that included the variables in the table in Multimedia Appendix 2 as covariates. Each patient was weighted by the inverse probability of being in their observed group, with the goal of balancing observable characteristics among the 3 groups within service. After propensity weighting, the balance among the characteristics in the groups is also given in Multimedia Appendix 2. In addition, after propensity weighting, within the service, comparisons of the ordinal PAM scores among groups were performed using robust weighted linear regression [30]. All analyses were performed using SAS version 9.4 (SAS Institute).

## Results

### Patient Characteristics and Participation

**Figure 2:**
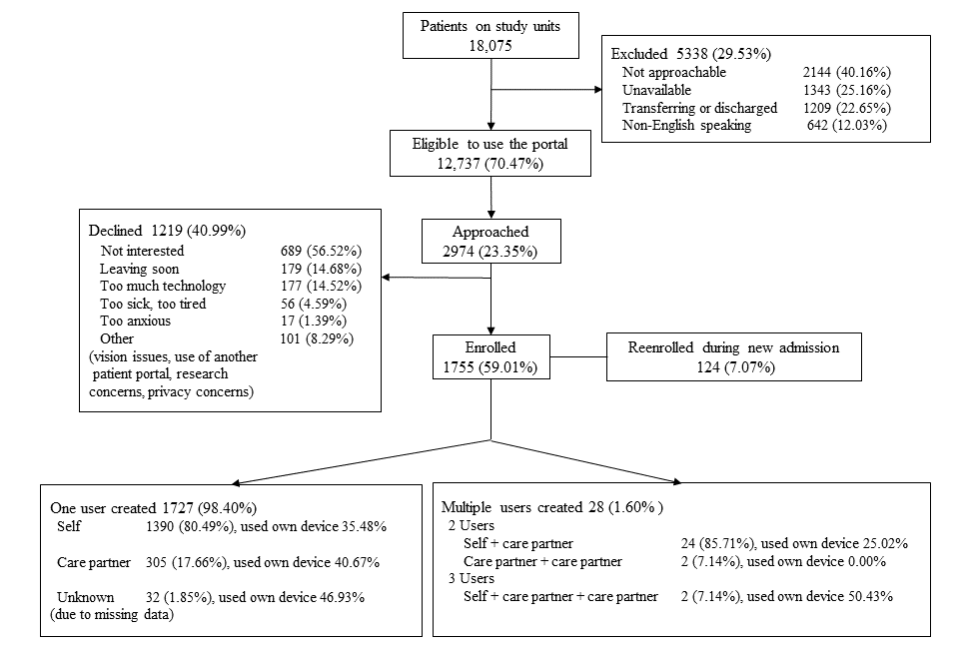
Recruitment flow diagram.

Of the 18,075 patients on our study units, 12,737 (12,737/18,075, 70.47%) patients met the inclusion criteria and 2974 (2974/12,737, 23.35%) patients were asked to use the portal (Figure 2). Of the patients who were approached by study staff, 1755 (1755/2974, 59.01%) patients were enrolled in the patient portal. The most frequent reasons patients cited for declining were that they were not interested (56.52%), were leaving the hospital soon (14.68%), it involved too much technology (14.52%), or they felt too sick or too tired (4.59%). Patients who enrolled to use the portal tended to be younger, less sick, less likely to have Medicare as an insurer, and were more likely to be registered for the Partners Healthcare enterprise ambulatory portal (Patient Gateway; Table 1). A total of 80.49% of users were patients whereas 19.51% were care partners, only 1.60% created multiple accounts. Of all the users, approximately 37% preferred to use their own devices over the tablet computers provided by the study team.

### Use of the Patient Portal

**Figure 3:**
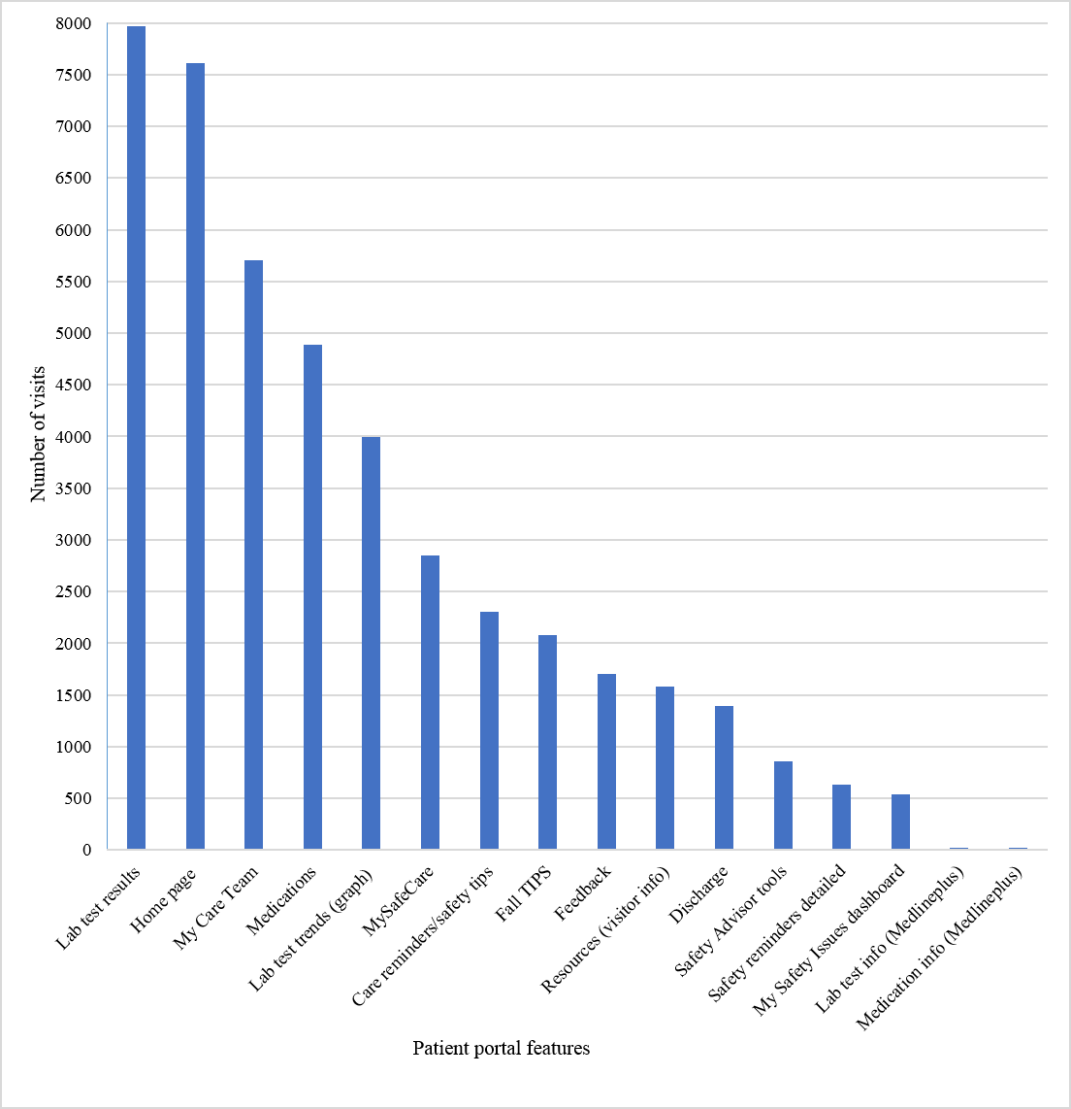
Patient portal use by feature. Some features were added after initial implementation, such as discharge was added on November 29, 2017, and the safety advisor (my safety issues dashboard) was added on January 1, 2018.

A total of 1637 patients and care partners were enrolled and received initial teaching on the portal. Approximately 65% of users did not use the portal beyond the first day, 20.28 used the portal for 2 days, and 14.66% used the portal for 3 or more days (Table 2). Most users (95.42%) accessed the portal from 1 to 4 days. On average, users logged into the portal at least once a day for 1.80 days (range: 1-32 days) and logged in 42.69% of the days that they had access during their hospitalization. The Test Results page was the most frequently visited, followed by My Care Team and Medications pages (Figure 3).

**Table 2:** Use of patient portal.

Usage measure		Portal users^a^, n=1637	Source of measure and source results		
Participated in teaching after enrollment, n (%)		1637 (100.00)	—^b^	—	—
Accessed portal (after initial teaching), n (%)		—	Grossman et al, 2017 [28], 10 (100)	Wilcox et al, 2016 [29], 20 (70)	Woollen et al, 2016 [7], 14 (86)
**Accessed portal for, n (%)**		—	—	—	—
	1 day only	1065 (65.06)	—	—	—
	2 days only	332 (20.28)	—	—	—
	3 or more days	240 (14.66)	—	—	—
**Accessed portal for, n (%)**		—			
	1-4 days	1562 (95.42)	Dalal et al, 2016 [27], 200 (84), n=239	—	—
	5-10 days	61 (3.73)	Dalal et al, 2016 [27], 39 (16), n=239	—	—
	>10 days	14 (0.86)	—	—	—
**Days used (logged in at least once per day)**		—	—	—	—
	Mean (SD)	1.80 (2.28)	Runaas et al, 2018 [22], 7.6 (6.3), n=20	—	—
	Range	1-32	—	—	—
**Days users had access (during hospitalization)**		—	—	—	—
	Mean (SD)	6.20 (7.24)	Runaas et al, 2018 [22], 21.3^c^, n-=20	Grossman et al, 2017 [28], 13.3^c^, n=20	Masterson-Creber et al, 2018 [20]^d^, n=426
	Range	1-66	Runaas et al, 2018 [22], 15-37, n=20	Grossman et al, 2017 [28], 4-38, n=20	—
**Percentage of days used during access period (during hospitalization)**		—	—	—	—
	Mean (SD)	42.69 (27.71)	Dykes et al, 2017 [5], Grossman et al, 2018 [11], *Brigham and Women’s Hospital patient-centered toolkit*,63^c^, n=194	—	—
	Range	1.5-100	—	—	—

^a^Number of patient portal users—there can be more than one portal user per patient enrolled. Total users n=1637 (patients with 1 user only, n=1602; 2 users only, n=16; and 3 users, n=1).

^b^No data.

^c^SD is not available.

^e^median=3.17.

### Patient and Caregiver Activation

There was an increase in PAM scores between the preintervention (usual care) group and the postintervention (safety dashboard and bedside safety display only) group on the neurology and medicine services but not on the oncology service (Table 3). On the oncology service, the mean PAM score decreased in the safety dashboard and bedside safety display only group, but the PAM scores in the patient portal group increased when compared with the usual care group. On the medicine service, the PAM scores increased in the safety dashboard and bedside safety display only group; however, there was a nonsignificant decrease in the patient portal group. All services showed an increase in mean PAM score in the patient portal group compared with the usual care group. Overall, after propensity weighting, the increase in PAM scores was statistically significant on the neurology and general medicine services. Although the sample size was too small to make formal comparisons, the observed CG-PAM score trends were similar to those seen with the PAM scores.

**Table 3:** Patient activation measure survey outcomes (propensity weighted).

Service	Usual care (preintervention^a^)		Safety dashboard + bedside safety display only (postintervention^b^)		Safety dashboard + bedside safety display + patient portal (postintervention)		*P* value
	PAM^c^-13, n (CG-PAM^d^)	Mean (95% CI)	PAM-13, n (CG-PAM)	Mean (95% CI)	PAM-13, n (CG-PAM)	Mean (95% CI)	
Neurology	124 (34)	61.3 (58.7-63.9)	127 (8)	64.8 (61.8-67.8)	251 (55)	71.4 (69.2-73.6)	<.001
Oncology	122 (14)	60.6 (57.6-63.0)	33 (4)	55.4 (45.9-64.9)	71 (6)	64.7 (60.3-69.2)	.14
Medicine	250 (21)	61.8 (59.9-63.8)	340 (8)	66.1 (64.3-67.9)	206 (6)	65.5 (63.0-68.1)	.01

^a^Surveyed September to November 2016.

^b^Surveyed December 2016 to May 2018.

^c^PAM: patient activation measure.

^d^CG-PAM: caregiver patient activation measure.

## Discussion

### Principal Findings

We implemented a patient portal over a large set of inpatient units spanning different services and found that patient activation scores improved in association with access to the tools in addition to an increase in the group with access to an acute care patient portal. Approximately 60% of the patients and care partners approached chose to enroll in the patient portal. This is much higher than the 18% enrollment rate we saw in our previous study with the Patient Centered Toolkit (also from our organization, implemented on a medical intensive care unit and an oncology unit) [5]. Despite successfully enrolling more patients than a previous study [5], many patients still chose not to participate. The leading reasons were that they were simply not interested, whereas others felt overwhelmed by unfamiliar technology or felt too sick to participate. Similarly, our previous study of the Patient Centered Toolkit found that 2 frequent reasons given for declining were personal preference or pending discharge [27]. Although we did not formally investigate the reasons that patients were not interested in using the portal, anecdotally we observed that many patients cited the use of other ambulatory patient portals such as Patient Gateway and did not want to use a second portal. Interestingly, this did not match our comparison of patient characteristics between those that enrolled and those that declined, which found that enrolled patients were more likely to be Patient Gateway users. This potential barrier to adoption was noted previously, along with a lack of access to the portal outside the hospital for care partners, which we also experienced, and it was recommended that access to an acute care patient portal be offered through ambulatory portals [27]. Providing these options in the future may help engage more patients and care partners in using similar technology.

We leveraged existing patient portal usage measures to describe the use of the acute care patient portal among enrollees to conduct comparisons. Overall, our portal usage was not as high as seen in previous studies (Table 2). Many patients did not use the patient portal beyond the first day, although there was a wide range of time periods that patients accessed the portal. We found that patients used the portal for fewer days during the access period than we reported previously with the Patient Centered Toolkit [5]. We were not able to distinguish between use on the first day during and after the initial teaching, and therefore were not able to directly compare with other research describing spontaneous use after initial teaching [7,28,29]. The average duration of portal use was also lower than what Runaas et al reported in their evaluation of an acute care patient portal, but it is possible that this is a result of the longer lengths of stay of their bone marrow transplant patient population [22]. We did not study patients’ perceptions of the portal or evaluate the usability of the portal during the intervention period. Patients may have found that the content was not useful to them or may have discovered usability or technical issues in accessing the portal; however, we met regularly with research staff who were enrolling patients on the clinical units and did not hear reports of usability issues from these staff.

We noted that the sample sizes for previous patient portal studies were much smaller than our study, and there was not enough data to conduct direct comparisons. Most of the previous studies were conducted as feasibility studies with small sample sizes. In contrast, our portal was implemented as a clinical trial, we approached close to 3000 patients and had limited resources for user support and follow-up. Although our study’s portal use demonstrated lower usage, we suspect one of the contributing factors may have been less engagement with users to encourage them to use the portal throughout their hospital stay. Our usage data imply that a one-time engagement with patients during portal enrollment was not enough to encourage continued use of the portal throughout the patients’ hospital stay. We have learned that ongoing support implemented into a clinical workflow may be key to successful implementation of an acute care patient portal and sustaining use beyond the research study.

Patients most often viewed their test results, care team members, and medication lists, similar to findings of other studies of acute care patient portals [27,28]. The tailored patient safety educational features that were unique to our patient-centered portal were not visited as often as we expected. We anticipated that these features could have impacted patient activation and safety. Although patients are interested in accessing their clinical information, we need a strategy to promote the use of additional portal features. Such strategies might include emphasizing the safety modules during teaching sessions or incorporating the use of the portal into formal patient education.

We found an association between patient activation and the use of patient-facing HIT tools in all study units, though not statistically significant in oncology. We hypothesized that the oncology service experienced different results because of organizational changes that occurred during our implementation period. In addition, many oncology patients were not able to participate in the PAM surveys and often had longer lengths of stay than patients on other services, contributing to the small sample size of oncology patients. This may have affected the results. The introduction of the bedside safety display on the neurology and medicine units may have led to the improved patient activation, and the use of an acute care patient portal in addition to the bedside safety display may have further improved patient activation. It is also possible that the patient groups surveyed had differing levels of activation at baseline; the patients who agreed to use the patient portal may have been more activated at baseline. This assumption was not always true based on our results—the bedside safety display group was more activated than the patient portal group in the medicine service. Overall, patient portals may help engage hospitalized patients and encourage active participation in care.

### Limitations

This study has several limitations: it was designed as a pre-post trial; it was conducted at a single academic medical center; the tools were independently designed; and it was only accessible to English-speaking patients or care partners. Additional studies are needed to assess the generalizability of our findings. We aimed to assess patient activation for patients using the acute patient portal; however, the other HIT tools such as the provider-facing safety dashboard and bedside patient display were implemented for all intervention groups and, therefore, we could not evaluate the effect of portal use on its own. We found an association between patient portal use and patient activation, but this may have been attributed to use and exposure to other components of the intervention.

In addition, our database had a technical issue causing some patient portal usage data to be lost; however, this was less than 10% of participants and other than length of stay, patient characteristics were similar to those of other portal users. Finally, our mobile app was only available on Apple smartphones, limiting some users that may have preferred to use their own mobile device.

### Conclusions

We found an association between the use of HIT tools, including a patient portal and patient safety display, and improved levels of patient activation in the inpatient setting. Such tools may be an effective mechanism to engage patients in their care and improve outcomes. Future study should continue to leverage existing usage metrics to assess patient portal use and focus on the impact of patient portals on specific outcome measures in the hospital setting.

## Appendix

Multimedia Appendix 1 Detailed description of patient portal features.

Multimedia Appendix 2 Demographics for patient activation measure survey (control factors).

## Abbreviations

AHRQ: Agency for Healthcare Research and Quality
BWH: Brigham and Women’s Hospital
CG-PAM: caregiver patient activation measure
EHR: electronic health record
HIT: health information technology
PAM: patient activation measure
PSLL: Patient Safety Learning Laboratory
