# Supplementary material for: Acute Care Patient Portal Intervention: Portal Use and Patient Activation
Source: J Med Internet Res. 2019 Jul 18;21(7):e13336. doi: 10.2196/13336 (PMC6670280; doi:10.2196/13336)
Supplement: Multimedia Appendix 1 [file jmir_v21i7e13336_app1.pdf]

Multimedia Appendix 1. Detailed description of patient portal features.

Patient Safety Learning Lab Patient Portal

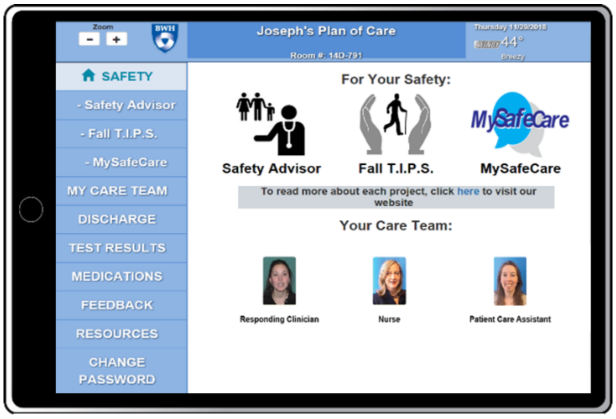

Home Page
First screen after log in. Displaying Safety Advisor, Fall TIPS, MySafeCare icons and preview of care team information. Includes link to research project website.

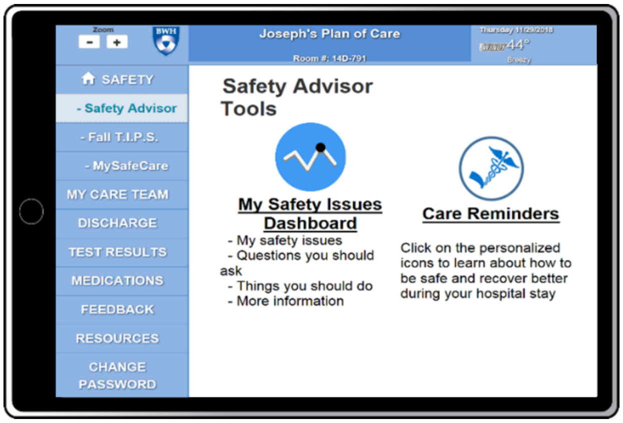

Safety Advisor Tools
Overview of Safety Advisor Tools: My Safety Issues Dashboard and Care Reminders.

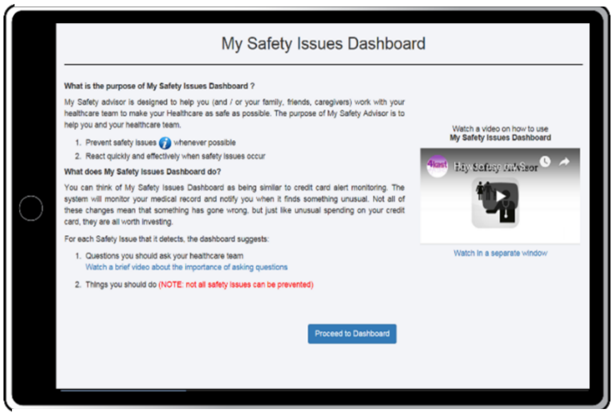

My Safety Issues Dashboard
Monitors safety metrics and displays graph of individual safety risk score. Alerts patients when something is unusual.

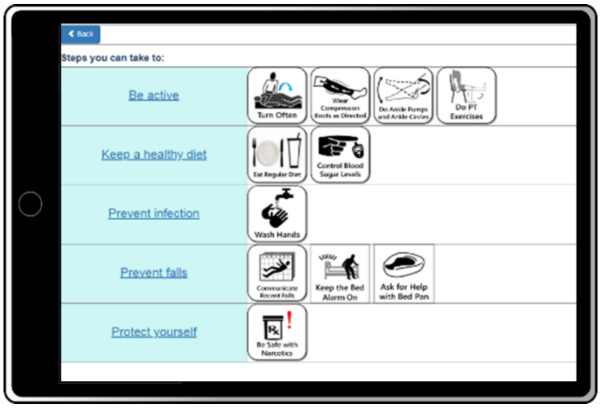

Care Reminders
Personalized safety reminders icons with links to additional information.

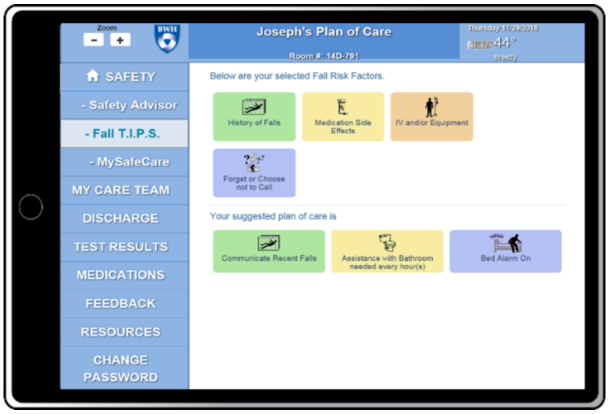

Fall T.I.P.S
Personalized fall prevention plan, displaying risk factor and plan of care icons.

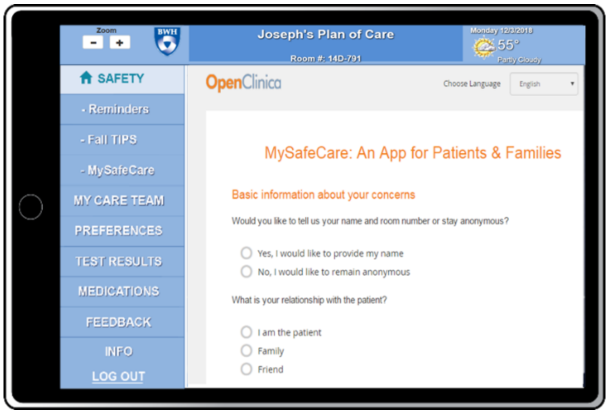

MySafeCare
Real-time reporting application to submit safety concerns or compliments.

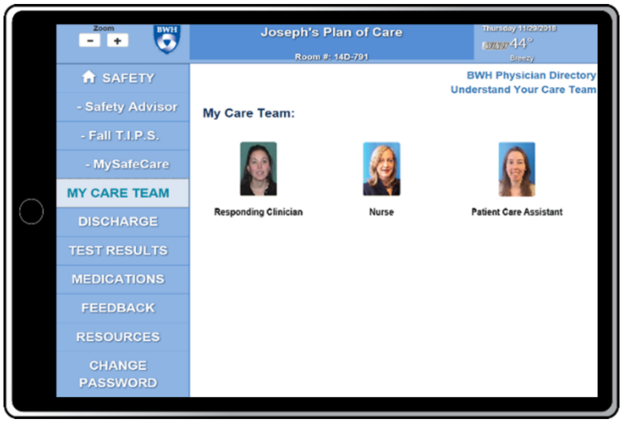

My Care Team
Names and photographs of patient's care team members. Updates with shift changes.

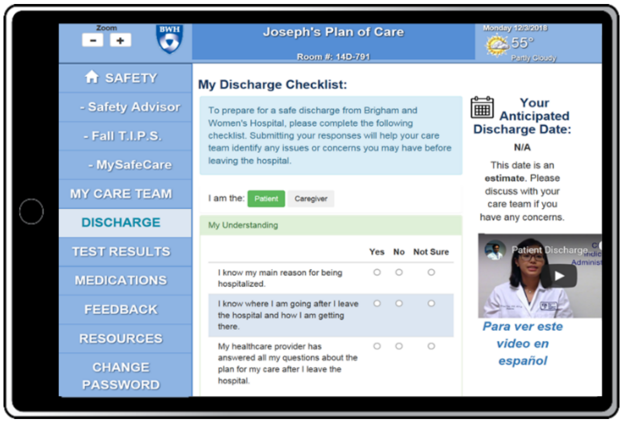

Discharge
Pre-discharge checklist, expected discharge date, and discharge preparedness video.

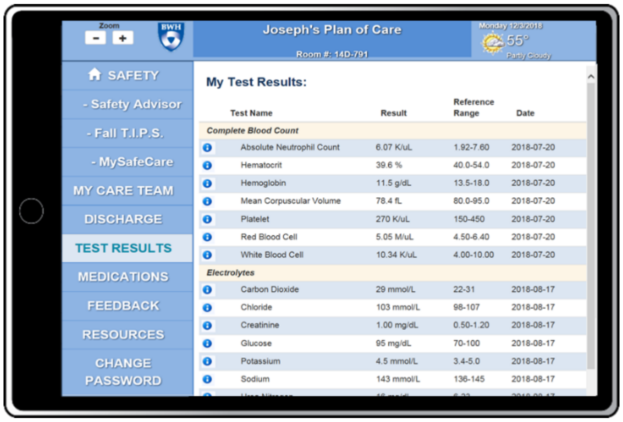

Test Results
List of most recent test results. Includes info button links to Medline Plus, and link to test trends graph.

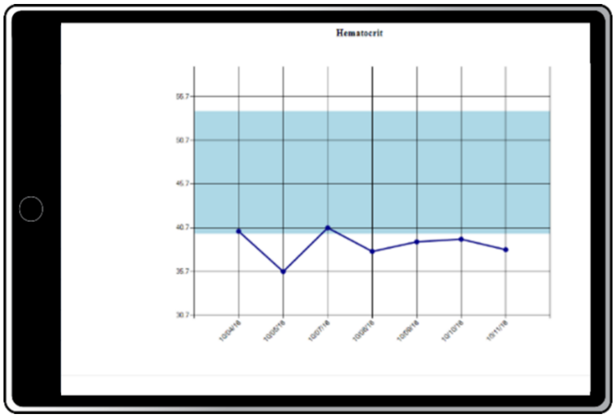

Test Trends Graph
Graph showing test result trends over time for a particular laboratory test.

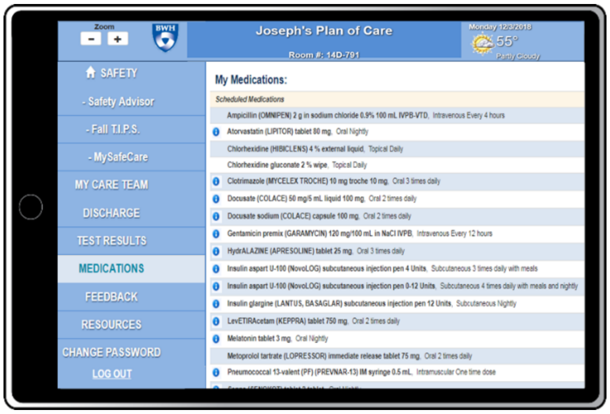

Medications
List of patient's hospital medications including scheduled and as needed medications. Includes info button link to Medline Plus.

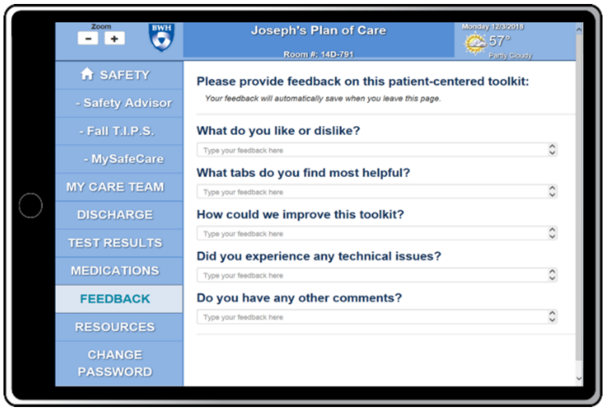

Feedback
Optional form for users to give feedback about their experience using the portal.

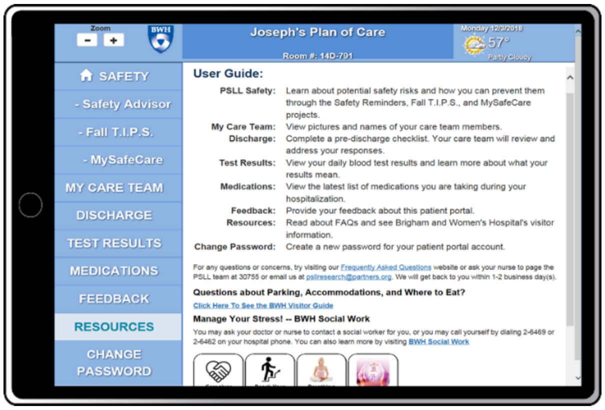

Resources
Additional information including brief 'User Guide', research team contact information, and hospital resources including: visitor information, social work, information for caregivers, Reiki information, breathing exercises, Medline Plus, Centers for Disease Control, and BWH Health Library.
