# Supplementary material for: Acute Care Patient Portal Intervention: Portal Use and Patient Activation
Source: J Med Internet Res. 2019 Jul 18;21(7):e13336. doi: 10.2196/13336 (PMC6670280; doi:10.2196/13336)
Supplement: Multimedia Appendix 2 [file jmir_v21i7e13336_app2.pdf]

Multimedia Appendix 2. Demographics for PAM survey (control factors).

		Before propensity weighting				After propensity weighting			
		**Usual care (preintervention)**	**Safety dashboard + bedside safety display only**	**Safety dashboard + bedside safety display + patient portal**	***P* value**	**Usual care (preintervention)**	**Safety dashboard + bedside safety display only**	**Safety dashboard + bedside safety display + patient portal**	***P* value**
**Neurology**		n=124	n=127	n=251	—[a]	n=124	n=127	n=251	—
Filled out by patients, n (%)		90 (72.6)	119 (93.7)	196 (78.1)	<.001	90 (72.6)	119 (93.7)	196 (78.1)	—
Gender, n (%)									
	Female	69 (56.1)	64 (50.8)	146 (58.9)	0.33	70 (55.8)	76 (56.9)	132 (56.1)	0.98
Age in years, mean (SD)									
		55 (16.8)	57.1 (17.6)	53.5 (15.0)	0.13	54.7 (1.7)	54.7 (1.6)	54.5 (1.0)	0.99
Race, n (%)									
	White	106 (88.3)	109 (88.6)	220 (90.5)	0.86	109 (88.3)	114 (87.2)	208 (89.7)	0.58
	Black	11 (9.2)	12 (9.8)	17 (7.0)		10 (8.2)	15 (11.8)	18 (8.0)	
	Other	3 (2.5)	2 (1.6)	6 (2.5)		4 (3.5)	1 (1.0)	5 (2.3)	
Ethnicity, n (%)									
	Hispanic or Latino	6 (5.6)	6 (6.0)	11 (4.9)	0.92	5 (4.2)	5 (5.0)	10 (4.7)	0.95
	Non-Hispanic or Latino	102 (94.4)	94 (94.0)	212 (95.1)		106 (95.8)	102 (201.1)	201 (95.3)	
Education, n (%)									
	High school or less	18 (15.3)	36 (29.8)	62 (25.3)	0.04	26 (21.4)	35 (26.9)	56 (23.9)	0.92
	Some college or associates	34 (28.8)	30 (24.8)	78 (31.8)		36 (29.4)	37 (28.5)	66 (28.3)	
	Bachelors, masters, or higher	66 (55.9)	55 (45.4)	105 (42.9)		60 (49.2)	57 (44.7)	111 (47.8)	
Primary language, n (%)									
	English	106 (87.6)	110 (90.9)	240 (95.6)	0.01	114 (91.5)	120 (93.5)	226 (94.4)	0.57
	Other	15 (12.4)	11 (9.1)	11 (4.4)		11 (8.5)	8 (6.5)	14 (5.6)	
**Oncology**		n=122	n=33	n=71	—	n=122	n=33	n=71	—
Filled out by patients, n (%)		108 (88.5)	29 (87.9)	65 (91.6)	0.77	108 (88.5)	29 (87.9)	65 (91.6)	—
Gender, n (%)									
	Female	79 (64.8)	19 (57.6)	40 (56.3)	0.46	77 (61.3)	22 (64.5)	37 (54.7)	0.61
Age in years, mean (SD)									
		60.42 (15.5)	60.64 (12.0)	55.17 (12.4)	0.03	59.2 (1.5)	59.0 (2.0)	57.6 (2.0)	0.79
Race, n (%)									
	White	104 (90.4)	28 (90.3)	61 (85.9)	0.85	108 (90.4)	29 (91.9)	58 (86.4)	0.62
	Black	5 (4.4)	2 (6.5)	5 (7.0)		5 (4.4)	2 (6.8)	4 (6.4)	

	Other	6 (5.2)	1 (3.2)	5 (7.0)		6 (5.2)	0 (0)	5 (7.2)	
Ethnicity, n (%)									
	Hispanic or Latino	9 (8.9)	1 (3.6)	2 (3.1)	0.36	7 (7.1)	1 (3.5)	1 (2.3)	0.33
	Non-Hispanic or Latino	92 (91.1)	27 (96.4)	61 (96.8)		98 (92.9)	28 (96.5)	57 (97.7)	
Education, n (%)									
	High school or less	26 (22.2)	6 (20.7)	9 (13.1)	0.22	23 (19.4)	5 (18.4)	14 (21.9)	0.93
	Some college or associates	34 (29.1)	4 (13.8)	19 (27.5)		34 (27.9)	8 (26.4)	14 (21.9)	
	Bachelors, masters, or higher	57 (48.7)	19 (65.5)	41 (59.4)		64 (52.8)	16 (55.2)	36 (56.3)	
Primary language, n (%)									
	English	107 (90.7)	31 (93.9)	67 (95.7)	0.42	112 (91.9)	32 (95.7)	59 (89.8)	0.67
	Other	11 (9.3)	2 (6.1)	3 (4.3)		10 (8.1)	1 (4.3)	7 (10.2)	
**Medicine**		n=250	n=340	n=206	—	n=250	n=340	n=206	—
Filled out by patients, n (%)		229 (91.6)	332 (97.7)	200 (97.1)	0.001	229 (91.6)	332 (97.7)	200 (97.1)	—
Gender, n (%)									
	Female	140 (56.5)	200 (59.0)	118 (58.1)	0.83	158 (58.3)	186 (57.4)	115 (59.1)	0.93
Age in years, mean (SD)									
		58.4 (17.4)	59.4 (18.4)	54.6 (17.6)	0.01	57.9 (1.2)	57.7 (1.1)	56.8 (1.2)	0.78
Race, n (%)									
	White	164 (71.3)	224 (70.1)	151 (78.2)	0.04	177 (69.9)	223 (73.8)	140 (75.6)	0.70
	Black	50 (21.7)	82 (25.9)	38 (19.7)		61 (24.2)	67 (22.2)	39 (21.0)	
	Other	16 (7.0)	11 (3.5)	4 (2.1)		15 (5.9)	12 (4.0)	7 (3.5)	
Ethnicity, n (%)									
	Hispanic or Latino	26 (12.8)	40 (12.8)	17 (9.3)	0.46	26 (11.5)	38 (12.9)	18 (10.2)	0.70
	Non-Hispanic or Latino	177 (87.2)	272 (87.2)	165 (90.7)		200 (88.6)	259 (87.2)	156 (89.8)	
Education, n (%)									
	High school or less	78 (32.6)	133 (40.3)	60 (30.0)	0.01	86 (32.9)	114 (36.1)	63 (32.9)	0.73
	Some college or associates	66 (27.6)	96 (29.1)	76 (38.0)		79 (30.2)	95 (30.2)	66 (34.4)	
	Bachelors, masters, or higher	95 (39.8)	101 (30.6)	64 (32.0)		97 (37.0)	106 (33.8)	63 (32.7)	
Primary language, n (%)									
	English	214 (88.8)	305 (92.4)	190 (94.5)	0.07	239 (90.8)	286 (90.8)	176 (91.4)	0.98
	Other	27 (11.2)	25 (7.58)	11 (5.5)		24 (9.2)	29 (9.2)	16 (8.6)	

[a]No data
